# Restoration of MARCK enhances chemosensitivity in cancer

**DOI:** 10.1007/s00432-020-03149-2

**Published:** 2020-02-13

**Authors:** Tim Wenzel, Thomas Büch, Nicole Urban, Ulrike Weirauch, Katrin Schierle, Achim Aigner, Michael Schaefer, Hermann Kalwa

**Affiliations:** 1 Rudolf-Boehm-Institut für Pharmakologie und Toxikologie, Medizinische Fakultät, Universität Leipzig, Härtelstraße 16-18, 04107 Leipzig, Germany; 2 Selbständige Abteilung für Klinische Pharmakologie Rudolf-Boehm-Institut für Pharmakologie und Toxikologie, Medizinische Fakultät, Universität Leipzig, Härtelstraße 16-18, 04107 Leipzig, Germany; 3 Institut für Pathologie, Medizinische Fakultät, Universität Leipzig, Liebigstraße 26, 04103 Leipzig, Germany

**Keywords:** Myristoylated Alanine-Rich C-Kinase Substrate, Phosphatidylinositol-4,5-bisphosphat, P-glycoprotein 1, Chemotherapy resistance, Colorectal cancer, ATP-binding-cassette (ABC) transporter

## Abstract

**Purpose:**

Increased ATP-binding-cassette (ABC) transporter activity is a major cause of chemotherapy resistance in cancer. The ABC transporter family member ABCB1 is often overexpressed in colorectal cancer (CRC). Phosphatidylinositol-4,5-bisphosphat (PI(4,5)P_2_)-dependent pathways are involved in the regulation of ABCB1 function. The protein Myristoylated Alanine-Rich C-Kinase Substrate (MARCKS) is a pivotal regulator of PI(4,5)P_2_ and inactivated in many CRC cancers via genetic deletion or hyperphosphorylation. Therefore, MARCKS may critically impact ABCB1.

**Methods:**

CRC samples as well as CRC cell lines were tested for a connection between MARCKS and ABCB1 via immunofluorescence and Western-blot analysis. ABCB1 function was studied via calcein influx assay under treatment with known ABCB1 inhibitors (verapamil, tariquidar) as well as the kinase inhibitor bosutinib. ABCB1 internalization and MARCKS translocation was analyzed via confocal microscopy exploiting the endocytosis inhibitors chlorpromazine and dynasore. Abundance of PI(4,5)P_2_ was monitored by intramolecular fluorescence resonance energy transfer (FRET). Reproductive cell survival was studied via colorimetric WST-1 and clonogenic assays in combination with exposure to the chemotherapeutics doxorubicin and 5-fuorouracil (5-FU).

**Results:**

We found increased ABCB1 expression in MARCKS negative CRC patient tumor samples and established CRC cell lines. Mechanistically, the reconstitution of MARCKS function via recombinant expression or the pharmacological inhibition of MARCKS phosphorylation led to a substantial decrease in ABCB1 activity. In CRC cells, bosutinib treatment resulted in a MARCKS translocation from the cytosol to the plasma membrane, while simultaneously, ABCB1 was relocated to intracellular compartments. Inhibition of MARCKS phosphorylation via bosutinib rendered cells more sensitive to the chemotherapeutics doxorubicin and 5-FU.

**Conclusions:**

Cells devoid of MARCKS function showed incomplete ABCB1 internalization, leading to higher ABCB1 activity enhancing chemoresistance. Vice versa our data suggest the prevention of MARCKS inhibition by reversing hyperphosphorylation or genomic restoration after deletion as two promising approaches to overcome tumor cell resistance towards chemotherapeutic ABCB1 substrates.

## Introduction

Development of resistance against chemotherapy is one of the main causes of tumor relapse (Borst and Schinkel 2013; Colabufo 2008; Katayama 2014). Therefore, further elucidating molecular mechanisms of chemoresistance, and interfering with these processes is critical to improve cancer therapy. In many solid tumors including colorectal carcinoma (CRC), the upregulation of ATP-binding cassette (ABC) transporters has been linked to resistance against various chemotherapeutics (Borst and Schinkel 2013; Colabufo 2008). ABCB1 (also known as P-gp, or as multidrug resistance protein 1, MDR1), is localized in lipid rafts and functions as an broad spectrum transporter, shuttling mostly lipophilic chemicals out of a cell, thereby minimizing the toxic effects of potentially harmful substances (Borst and Schinkel 2013; Lavie et al. 1998; Mercier 2012). However, underlying mechanisms of the (up-)regulation of ABCB1 function remain to be uncovered, but are pivotal for overcoming primary or secondary chemoresistance.

A number of reports have demonstrated that phosphatidylinositol-4,5-bisphosphate (PI(4,5)P_2_)-dependent pathways are critically involved in the modulation of ABCB1 function (Echard 2012; Kobori et al. 2014). Therefore, we postulated that the PI(4,5)P_2_ storage protein Myristoylated Alanine-Rich C-Kinase Substrate (MARCKS) impacts ABCB1 regulation. MARCKS has been implicated in cancer, since its loss is associated with unfavorable patient outcome (Bickeböller et al. 2015; Chen et al. 2014, 2015; Yang et al. 2015). The main feature of MARCKS is the formation of a storage compartment for PI(4,5)P_2_. Under physiological conditions, MARCKS resides at the plasma membrane. Due to its electrostatic properties MARCKS binds and sequesters PI(4,5)P_2_. This reduces the accessible lipid pool and by this means regulates signal transduction. Upon phosphorylation by protein kinase C (PKC) or the oncogenic tyrosine kinase c-Abl, MARCKS dissociates from the membrane, releasing PI(4,5)P_2_ for interaction with other target proteins.

Previously, two independent groups (Bickeböller et al. 2015; Chen et al. 2015) found that roughly 40% of tested colon carcinoma samples lack MARCKS expression. This was confirmed by the data from the Human Protein Project (Thul et al. 2017; Uhlen et al. 2017; Uhlén et al. 2015). According to these analyses, 6 out of 12 tested tumor samples are devoid of MARCKS. In cases, where MARCKS is detectable, a wide range of expression levels has been observed, in contrast to uniformly high MARCKS levels in healthy tissue. Notably, however, the activity of MARCKS depends not only on its presence, but also on its phosphorylation state (Chen et al. 2014). In its phosphorylated form, MARCKS is relocalized into the cytosol and, as a consequence, the main MARCKS function (sequestration of PI(4,5)P_2_) is impaired. Of note, loss of MARCKS function either by deletion or hyperphosphorylation was found to correspond with a worse patient outcome and enhanced therapeutic resistance (Bickeböller et al. 2015; Chen et al. 2014, 2015; Yamaguchi et al. 2006; Yang et al. 2015). Concomitantly, when screening various well-established CRC cell lines for expression and phosphorylation state, MARCKS deficiency (e.g., LoVo cells) or hyperphosphorylation (inactivation despite large MARCKS amounts; e.g., HT-29 cells) were found as well (Bickeböller et al. 2015).

Based on these findings, we used HT-29 and LoVo cells as models to investigate the interplay between ABCB1 and MARCKS. Here we employed the ectopic reintroduction of MARCKS phosphorylation mutants S4A (PI(4,5)P_2_ signaling specific 4 serines are mutated) and S4D (phosphomimetic of PI(4,5)P_2_ signaling specific 4 serines) in LoVo cells. Additionally we could show previously that MARCKS phosphorylation largely depends on the function of the tyrosine kinases c-Abl and Src. We tested compounds related to this signaling pathway and explored the dual c-Abl/Src tyrosine kinase inhibitor bosutinib (SKI606) as a potent blocker of MARCKS phosphorylation (Jin et al. 2012; Kalwa et al. 2012, 2014; Kalwa and Michel 2011). Thus, beyond the ectopic overexpression of different MARCKS mutants in LoVo cells, the treatment of HT-29 cells with bosutinib provides a tunable system of MARCKS function to analyze the potential crosstalk between ABCB1 and MARCKS in the context of resistance against chemotherapeutic agents.

## Methods

### Chemicals and reagents

HT-29 and LoVo cells were from ATCC (American Tissue Type Culture Collection, USA). Both cell lines were cultured in IMDM (Iscove’s Modified Dulbecco’s Medium, Sigma-Aldrich), supplemented with 10% FBS (Fetal Bovine Serum, Gibco), penicillin–streptomycin, and l-glutamine at 37 °C and 5% CO_2_ under moist atmosphere. Transfections were performed using Lipofectamine 2000 (Invitrogen) according to manufacturer’s protocol. Stable lines of HT-29 and LoVo cells recombinatly expressing MARCKS-eGFP or ABCB1-eGFP were selected in media supplemented with 0.5 mg/mL G418 (Sigma-Aldrich, Taufkirchen, Germany).

Primary antibodies directed against MARCKS (#5607), phospho-MARCKS (#8722), ABCB1 (#13342), and vinculin (#13901) were from cell signaling technology (Boston, USA). Calcein-AM was from AAT Bioquest (Sunnyvale, CA), WST-1 reagent was from Roche Diagnostics GmbH (Mannheim, Germany). 5-FU was provided from local compounding pharmacy. All other reagents were from Sigma-Aldrich (Taufkirchen, Germany).

All EV-fluorescence resonance energy transfer (FRET) biosensor plasmids were kind gifts of Michiyuki Matsuda. MARCKS-HA and MARCKS ΔED-HA were a gift from Hendrik Bläker. ABCB1-GFP was a gift from Tounsia Aït Slimane. MARCKS mutands S4A and S4D were a kind gift of Wanli Liu.

### Calcein uptake assay

Cells were seeded in 24-well plates (Greiner) and incubated for 24 h. Cells were washed and incubated with modulators, e.g., bosutinib (5 µM) and verapamil (50 µM), or their solvent in Iscove’s Modified Dulbecco’s Medium (IMDM) for 15 min at 37 °C. Then, calcein-AM (AAT Bioquest, Boston) dissolved in dimethyl sulfoxide (DMSO, stock concentration of 2 mM) was added at a final concentration of 1 µM. Afterwards cells were incubated in a microplate reader (PolarStar Omega, BMG Labtech, Ortenburg, Germany) at a temperature of 37 °C. Calcein fluorescence was excited at 485 nm and detected at 520 nm. Orbital measurements of a circle with a diameter of 8 mm were performed and averaged to compensate for possible inhomogeneities of cell growth.

### WST-1 assay

HT-29 cells were seeded in a 96-well plate (Greiner 96 W F-Bottom), sparing the external wells which were exclusively filled with culture medium (IMDM, Sigma-Aldrich). After 24 h the viability of 12 wells filled with cells was measured using a colorimetric WST-1 assay (water-soluble tetrazolium), according to manufacturer’s protocol. In brief, 10 µL of WST-1 (Roche Diagnostics GmbH, Mannheim, Germany) were added to the cells, incubated for 30 min and absorbance was measured at 450 nm with a reference wavelength of 850 nm with the Polarstar Omega microplate reader. After the agents were added to the other wells (5-FU 1 µM, bosutinib 0.5 µM), incubated and measured as described above at days 1 and 4.

### Clonogenic survival assay

In addition to the WST-1 assay, effects of 5-FU or bosutinib exposition on cell viability were monitored using the clonogenic survival assay. In brief, 5 × 10^5^ cells growing in IMDM in 25 cm^2^ cell culture flasks were treated with the respective agent, i.e., vehicle, 0.5 µM 5-FU, 0.5 µM bosutinib, or a combination of 5-FU and bosutinib (0.5 µM each) for 48 h. Afterwards, cells were trypsinized and counted using a hemocytometer. 1000 cells per condition were re-seeded into a 6-well plate and incubated in normal culture medium (without any further treatment) for 7 days. Thereafter, the medium was aspirated. The colonies were gently washed with PBS, and then stained by use of 0.5% (w/v) methylene blue in a 1:1 mixture (v/v) of ethanol and water. The colonies were incubated for 15 min with the staining solution, then gently washed with deionized H_2_O and dried at room temperature.

### Western blot

To prepare samples for western blotting, cells were harvested with a cell scraper, washed with PBS and homogenized with a lysis buffer, containing 150 mM NaCl, 50 mM Tris, 1 mM EDTA, 1% NP-40, 0.25% sodium deoxycholate, 1 mM NaF, 1 mM sodium orthovanadate, 1 µM phenylmethylsulfonyl fluoride, 1 µg/mL aprotinin, 1 µg/mL leupeptin, and 1 µg/ml pepstatin. Equal amounts were lysed in Laemmli buffer, and loaded to 10% SDS-PAGE. Separated proteins were electroblotted onto nitrocellulose membranes, and membranes were blocked for 1 h in 5% nonfat dry milk dissolved in TBS (20 mM Tris pH 7.4, 137 mM NaCl), supplemented with 0.5% Tween-20. Antibodies were incubated in 1% BSA Tris-buffered saline and 0.5% Tween-20 overnight. The next day, the membrane was washed, followed by incubation with secondary anti-rabbit peroxidase-conjugated antibodies (Sigma-Aldrich; Germany; 1:5000) for 3 h and analyzed via a cooled CCD camera using a chemiluminescence reagent SuperSignal West Pico Kit (Pearce).

### Fixed tissue imaging

For analyses of fixed tissues, paraffin-embedded samples were mounted on glass slides. After paraffin removal and epitope unmasking treatment (Dako retrieval solution), slides were rinsed with PBS, permeabilized in 0.1% Triton X-100 in PBS for 5 min, and blocked with 10% goat serum in PBS for 1 h. Incubations with primary antibodies were performed in blocking solution at 4 °C overnight. After washing with PBS, slides were incubated with secondary antibody conjugated to fluorescent dye (Alexa Fluor 488 anti-rabbit IgG) in blocking solution for 2 h at room temperature. Images were acquired with an inverted LSM 510 META confocal microscope (Carl Zeiss).

### Confocal imaging

Coverslips with adherently growing HT-29 cells transfected with cDNAs encoding MARCKS-eGFP or ABCB1-eGFP were placed in a chamber containing HBS and mounted onto the stage of an inverted LSM 510 META confocal microscope (Carl Zeiss). Imaging was performed with excitation at 488 nm and emission was filtered with a 500 nm long-pass filter. A Plan-Apochromat 100X/1.46 objective (Carl Zeiss) was used, and pinholes were set to yield optical slices with a thickness of 0.6–0.8 µm. After an incubation of 5 min, visual fields were selected, and the MARCKS and ABCB1 distribution was imaged over time. Translocation was triggered by adding bosutinib (0.5 µM final concentration) to the bath solution. For statistical analysis, regions of interest were defined in cytoplasmic areas of single cells. Finally, data was normalized to initial cytosolic GFP intensity.

### FRET imaging

Monitoring of fluorescence resonance energy transfer (FRET) biosensors was performed using methods described in detail previously (Aoki et al. 2013; Kalwa et al. 2012, 2014; Kalwa and Michel 2011). In brief, HT-29 cells were transfected with plasmids encoding FRET biosensors as indicated. After 24 h, the cells were seeded onto 24-mm glass plates, pretreated with inhibitors as indicated, and subjected to excitation of CFP-PH at 425 ± 10 nm; emission was collected at 475 ± 10 nm (CFP) and 540 ± 10 nm (YFP) using the AHF FRET-CFP/YFP filter single-band set. A series of fluorescence images were taken at 30 s time intervals before and after drug treatments. Visualization and analysis was performed using ImageJ software.

### Statistical analyses

All experiments were performed at least three times. Data were assessed using ANOVA with the Student–Newman–Keuls post hoc test or Student’s *t* test with Welsh’s correction as appropriate. Data are expressed as mean ± SEM. P values below 0.05 were considered significant.

## Results

### Colon carcinoma cells show reduced MARCKS expression or enhanced MARCKS phosphorylation

The present investigation is based on the hypothesis that MARCKS—via its ability to bind phosphatidylinositol 4,5-bisphosphate (PI(4,5)P_2_)—affects the function of the ABCB1 transporter.

**Fig. 1:**
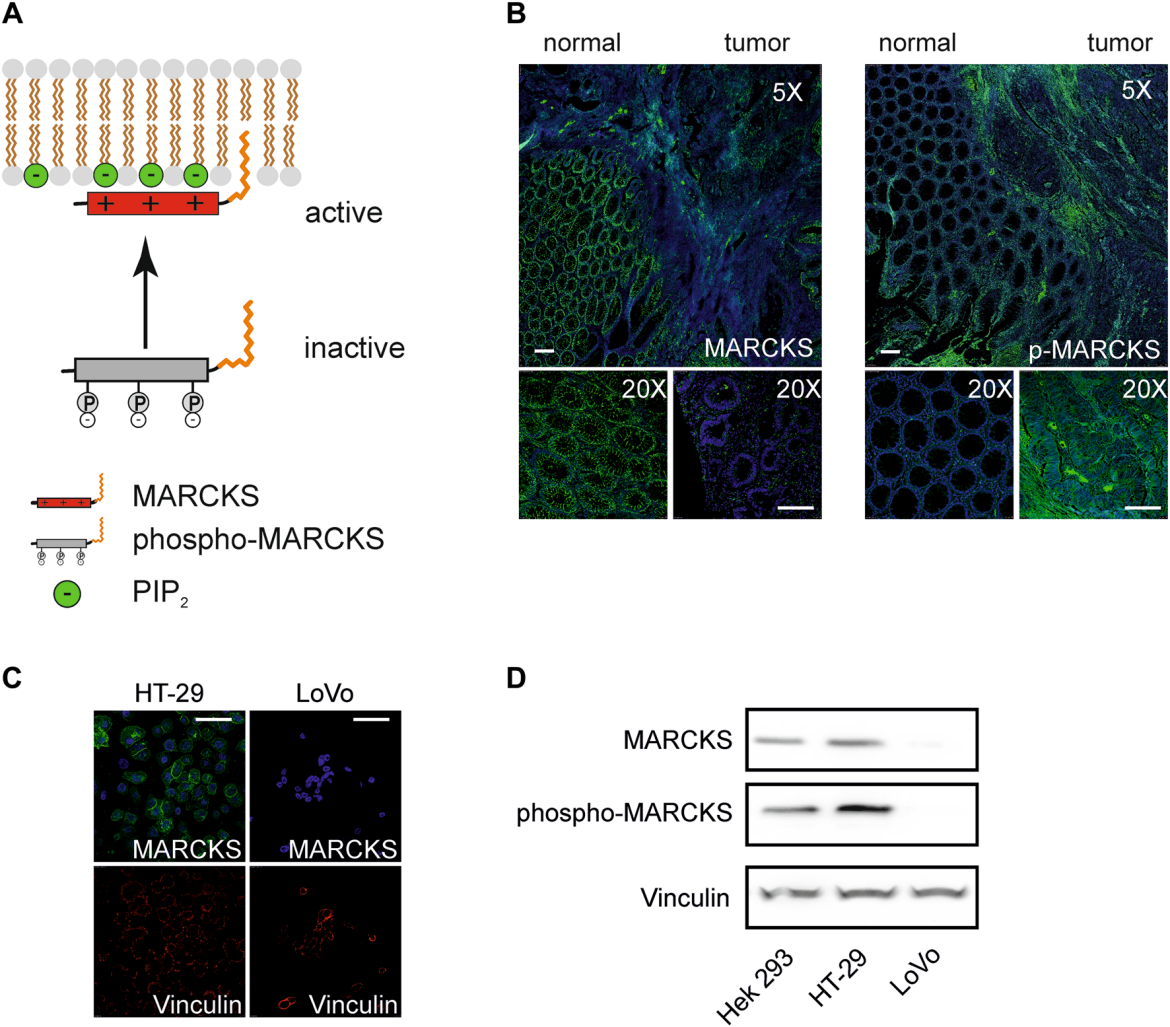
MARCKS phosphorylation in CRC tissue and CRC cell lines. **a** Phosphorylation-dependent PIP2 sequestration by MARCKS. Subcellular location of MARCKS and its binding ability for PIP2 is regulated via its phosphorylation status. MARCKS kinases (e.g., PKC or c-Abl) induce translocation of the protein to the cytoplasm and by this means impair PIP2 sequestration. **b** MARCKS expression in CRC. Shown are representative photomicrographs of human CRC tissue preparations that were fixed, paraffin-embedded, and stained with antibodies as indicated. Depicted is a lower magnification overview at the border between normal and cancerous tissue as well as higher magnification pictures of individual areas (left panel) MARCKS or phosphorylated MARCKS protein (right panel) expression is visualized in green using Alexa 488 conjugated secondary antibodies. Nuclei were stained in blue with DAPI. Pictures were obtained by confocal imaging. Bar indicates 100 µm. **c** and **d** MARCKS expression in CRC cell lines LoVo and HT-29. Two CRC cell lines selected for either hyperphosphorylation (HT-29) or deletion of MARCKS protein (LoVo) were fixed and stained with antibodies against MARCKS (green) and the protein Vinculin (red) as control. Nuclei were stained with DAPI (blue). Pictures were obtained by confocal imaging. Bar indicates 100 µm. **d** Representative examples from western blots of HEK 293 control cells, HT-29 and LoVo cells. Cells were treated lysed, harvested, blotted and probed with antibodies against phospho-MARCKS, MARCKS and vinculin serving as a loading control

In this context, it is noteworthy that the PI(4,5)P_2_-binding function of MARCKS is regulated by its phosphorylation state: Whereas non-phosphorylated MARCKS is associated with the plasma membrane and interacts with PI(4,5)P_2_, phosphorylated MARCKS (phospho-MARCKS) is translocated to the cytoplasm and does not interfere with PI(4,5)P_2_ (Fig. 1a). Thus, with respect to its role in PI(4,5)P_2_-binding (and irrespective of other, PI(4,5)P_2_-independent MARCKS functions) phospho-MARCKS can be designated as inactive, whereas non-phosphorylated MARCKS acts as the active variant (Fig. 1a).

Of note, lack of MARCKS expression in colorectal cancer (CRC) has been associated with a more aggressive tumor phenotype and unfavorable prognosis (Chen et al. 2014, 2015; Rombouts et al. 2013), suggesting a tumor suppressor function of MARCKS (Rombouts et al. 2013). Thus, if the antitumor effects of MARCKS are dependent on its ability to interact with PI(4,5)P_2_, CRC cells with hyperphosphorylated (inactive) MARCKS should behave like CRC cells with absent MARCKS. Therefore, we performed immunohistochemistry analyses of CRC tumor samples from nine patients to establish the presence of phosphor-MARCKS in CRC. In fact, MARCKS staining (total MARCKS) was absent in three tumor samples and markedly reduced in another three samples in contrast to strong MARCKS staining in adjacent normal tissue (Fig. 1b, left). This finding is in line with the aforementioned postulate of a tumor-suppressive role of MARCKS in CRC. However, in three further samples increased phospho-MARCKS signals were detectable as compared to the adjacent normal tissue (Fig. 1b, right). This provided the rationale to elucidate the potential role of phosphorylated MARCKS in CRC.

**Fig. 2:**
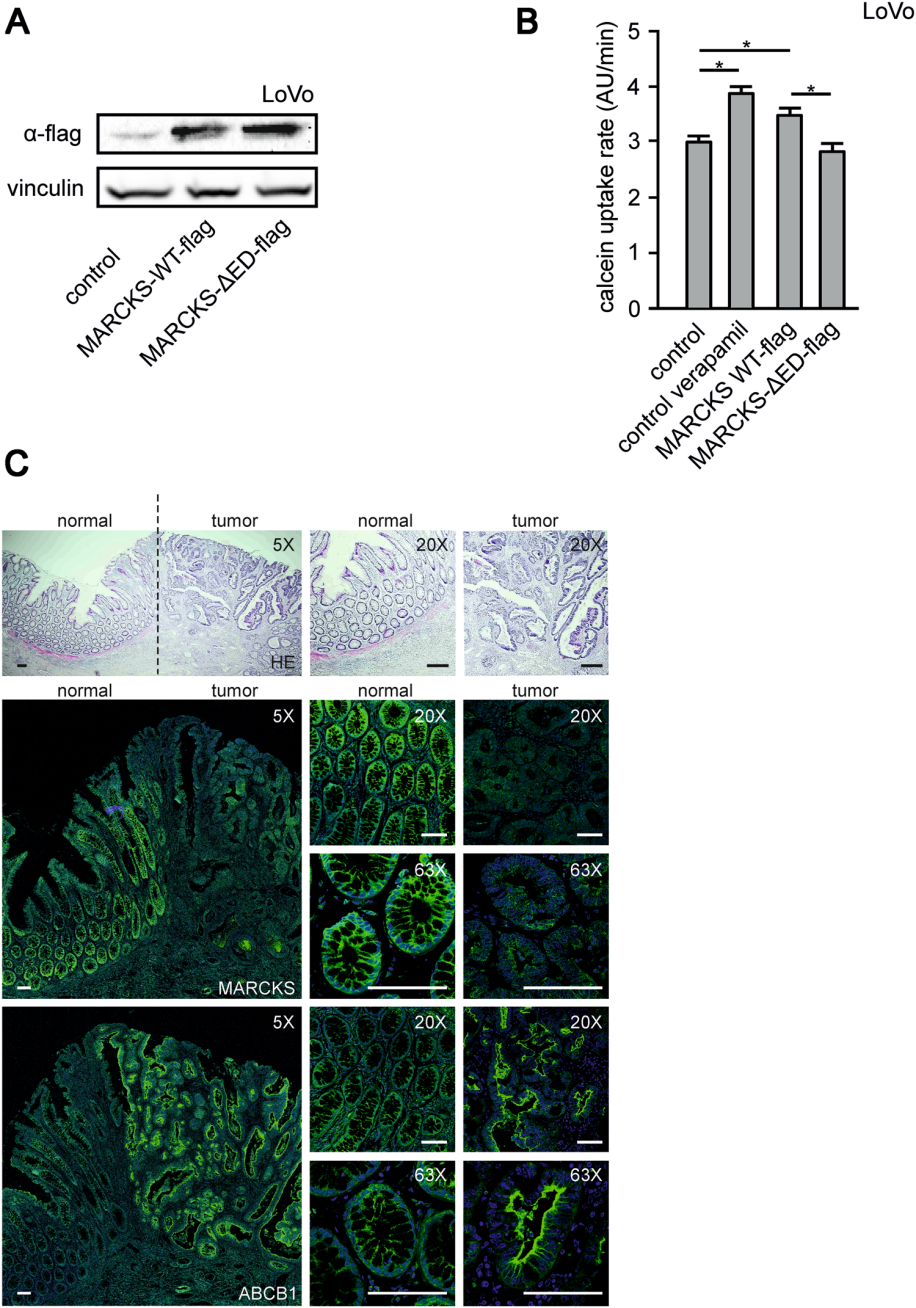
MARCKS and ABCB1 expression and function in CRC tissue and CRC cell lines. **a** and **b** Restoration of MARCKS expression in LoVo cells. **a** Representative western blot is shown for LoVo cells transfected with cDNA´s encoding flag-tagged, functional MARCKS or a MARCKS-Flag effector domain mutant lacking the PIP2-binding function of MARCKS. **b** Calcein assay of LoVo cells treated with verapamil or transfected with cDNA’s encoding different MARCKS constructs as indicated. **c** Expression of MARCKS and ABCB1 in CRC. Representative photomicrographs are shown of human CRC tissue preparations that were fixed, paraffin-embedded, and stained with haematoxylin and eosin (upper panel). Depicted is a lower magnification overview between normal and cancerous tissue as well as higher magnification pictures. The lower panel shows stainings at different magnifications of consecutive slices treated with antibodies directed against the ABCB1 protein or MARCKS and Alexa 488 (green) conjugated secondary antibodies as indicated. Nuclei were stained with DAPI (blue). Pictures were obtained by confocal imaging. Bar indicates 100 µm

For this purpose, we used in the following investigations the two well-established human CRC model cell lines LoVo and HT-29 (Fig. 1c). These two cell lines are particularly suitable for the functional characterization of MARCKS in the CRC context, since LoVo cells completely lack MARCKS expression owing to a genomic deletion (Bickeböller et al. 2015; Rombouts et al. 2013) and HT-29 cells show heavily phosphorylated MARCKS (Bickeböller et al. 2015) (Fig. 2c, d).

### Evidence for a connection between MARCKS expression and ABCB1 function using MARCKS-negative LoVo cells

To further test whether MARCKS negatively affects ABCB1 we used MARCKS-negative LoVo cells and introduced wild-type MARCKS with a flag tag (MARCKS-WT-flag) or effector domain-negative flag-tagged MARCKS (MARCKS-ΔED-flag), which does not bind PI(4,5)P_2_ (Fig. 2a).

Expressions of wild-type MARCKS, but not of inactive, effector domain-mutated MARCKS increased the uptake of the ABCB1 substrate calcein into LoVo cells, indicating a reduced function of the ABCB1 efflux pump (Fig. 2b). This effect of MARCKS expression on calcein uptake was comparable with the impact of the well-established ABCB1 blocker verapamil (Fig. 2b), indicating the potential relevance of this mechanism.

Moreover, MARCKS and ABCB1 expression were inversely correlated in six CRC tumor samples with reduced or absent MARCKS expression and in adjacent normal tissue: Normal tissue showed high MARCKS and low ABCB1 expression and tumor tissue displayed low MARCKS and high ABCB1 expression (Fig. 2c). Of note, this inverse correlation of MARCKS and ABCB1 expression was also observed in three tumor samples with highly phosphorylated MARCKS (data not shown), suggesting that the phosphorylation status of MARCKS is relevant for its impact on ABCB1 function.

**Fig. 3:**
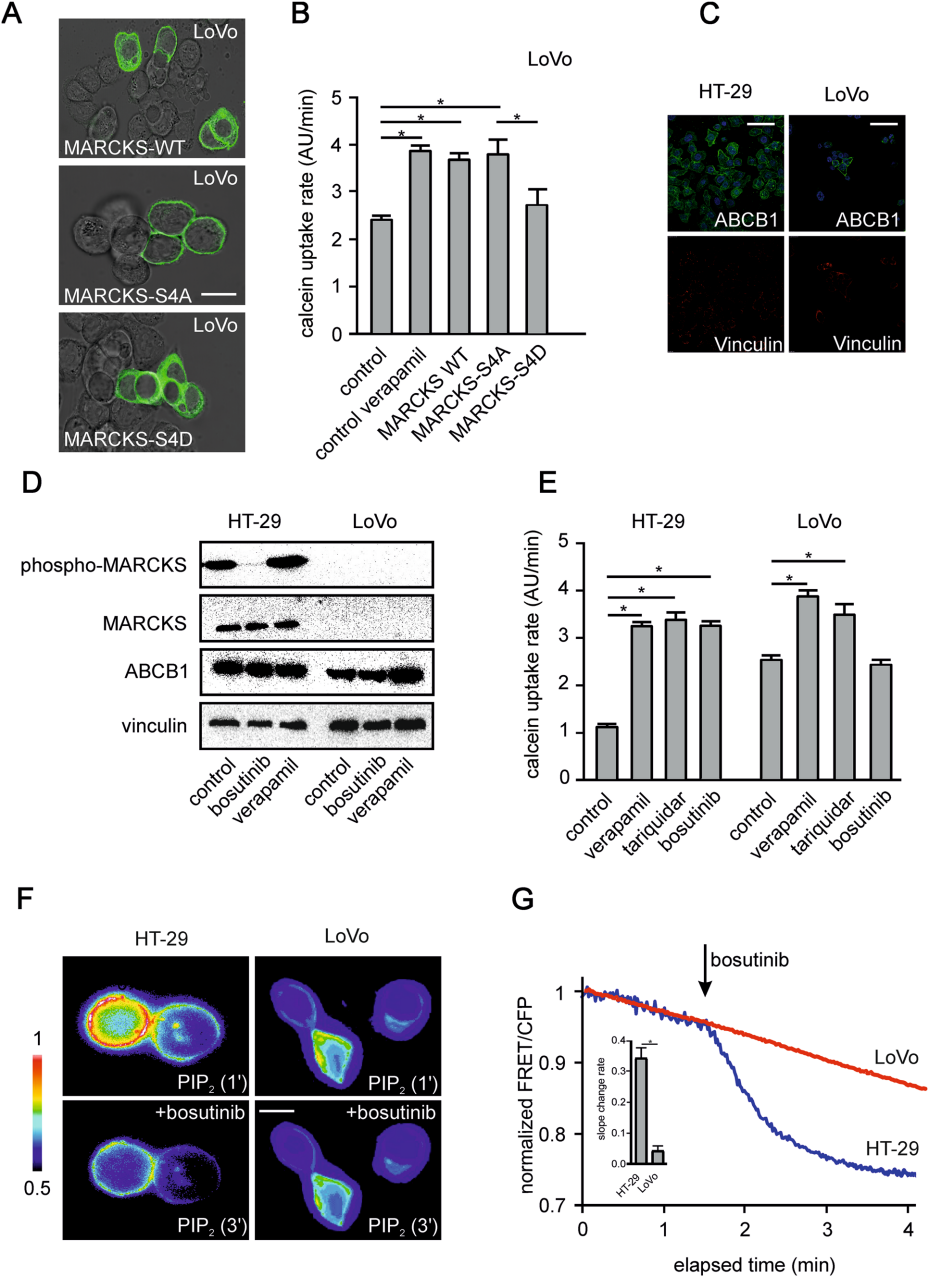
Phosphorylation of MARCKS and abundance of PIP2 in HT-29 and LoVo cells. **a** shows representative images of LoVo cells transfected with cDNA’s encoding the fluorescence-tagged (GFP) MARCKS proteins MARCKS-WT, MARCKS S4A and MARCKS S4D as indicated and analyzed via confocal imaging. **b** Calcein assay of LoVo cells treated with verapamil or transfected with cDNA´s of MARCKS-WT, or the phosphorylation mutants MARCKS S4A and MARCKS S4D as indicated. All data are aggregates from at least three independent experiments. **c** The CRC cell lines selected for either hyperphosphorylation (HT-29) or deletion of MARCKS protein (LoVo) were fixed and stained with antibodies against ABCB1 (green) and the protein Vinculin (red) as control. Nuclei were stained with DAPI (blue). Pictures were obtained by confocal imaging. Bar indicates 100 µm. **d** Representative examples from western blots of HT-29 and LoVo cells treated as indicated. Cells were incubated for 15 min with bosutinib or verapamil as depicted, lysed, harvested, blotted and probed with antibodies against phospho-MARCKS, MARCKS, ABCB1 and vinculin. Shown in **e** is pooled slope data of calcein assays of HT-29 or LoVo cells as indicated. Cells were treated with the ABCB1 inhibitors verapamil or tariquidar or bosutinib. **f** Changes in PI(4,5)P_2_ abundance were measured via FRET. **g** HT-29 and LoVo cells were transfected with a cDNA encoding the radiometric single chain FRET sensor PiPi(4,5) specific for PI(4,5)P_2_ and treated with bosutinib as indicated. **g** Changes in PI(4,5)P_2_ abundance were tracked via fluorometric microscopy for 5 min in the presence or absence of bosutinib. **g** Shows the calculated change in slope (FRET ratio) before and after the addition of bosutinib. Shown are pooled data of at least three experiments

Based on these findings, we next made use of a MARCKS mutant with absent phosphorylation sites (MARCKS-S4A) and a MARCKS mutant with phospho-mimicking mutations (MARCKS-S4D). In cells expressing MARCKS-S4A, the inhibition of MARCKS function via phosphorylation (cf. Fig. 1a) is abrogated. In contrast, the MARCKS-S4D mutant behaves like inactive, hyperphosphorylated MARCKS. In line with these assumptions, LoVo cells expressing wild-type MARCKS showed a mixed subcellular distribution of MARCKS both in the cytoplasm and in association with the plasma membrane (Fig. 3a, top panel). In contrast, the constitutively active MARCKS-S4A mutant was only expressed at the plasma membrane (Fig. 3a, middle panel) and the inactive (phospho-mimicking) MARCKS-S4D mutant was translocated into the cytoplasm (Fig. 3a, lower panel).

Using these MARCKS constructs, we again tested for the effect of calcein uptake in LoVo cells (Fig. 3b). In line with the findings with flag-tagged MARCKS (cf. Fig. 2b), both wild-type and constitutively active MARCKS (MARCKS-S4A) increased calcein uptake (suggestive of an ABCB1 inhibition), whereas the inactive MARCKS-S4D mutant had no effect on calcein accumulation (Fig. 3b).

### Role of MARCKS phosphorylation for regulation of ABCB1 function

In HT-29 cells with hyperphosphorylated MARCKS showing a functional inactivation of MARCKS (cf. Fig. 1a) this inhibition represents a potential therapeutic option to modulate ABCB1 activity. First we tested for the expression of ABCB1 in HT-29 and LoVo cells via immunostaining and Western blot (Fig. 3c and d).

As previously shown (Jin et al. 2012; Kalwa et al. 2012, 2014; Kalwa and Michel 2011), MARCKS phosphorylation in endothelial cells is reduced by the tyrosine kinase inhibitor bosutinib. Bosutinib also inhibited MARCKS phosphorylation in HT-29 cells (Fig. 3d). As expected, LoVo cells showed no MARCKS expression (Fig. 3d). In the functional calcein assay both in HT-29 and LoVo cells the accumulation of calcein over time was accelerated upon addition of the well-established ABCB1 inhibitors tariquidar (0.1 µM) and verapamil (50 µM) (Fig. 3e) and both cell lines have detectable levels of ABCB1 expression. Notably, bosutinib (5 µM) treatment increased calcein accumulation in HT-29 cells in a similar manner as the direct ABCB1 blocker verapamil (3.2-fold increase in calcein uptake rate, *p* = 0.002; *n* = 35; Fig. 3e). In contrast, in LoVo cells, which are devoid of MARCKS (Fig. 3d), bosutinib did not exert any effect on calcein uptake, despite intact ABCB1 function as indicated by the verapamil and tariquidar positive controls (Fig. 3e). This indicates a direct role of MARCKS in ABCB1 regulation.

To assess the role of PI(4,5)P_2_, we used a FRET biosensor (Aoki et al. 2013) for PI(4,5)P_2_. Treatment of HT-29 cells with bosutinib markedly decreased the level of free PI(4,5)P_2_ in the plasma membrane, whereas bosutinib had no effect in MARCKS-negative LoVo cells (Fig. 3f, g). Of note, bosutinib treatment only affected the abundance of the MARCKS binding partner PI(4,5)P_2_, but not the level of PI(3,4)P_2_, which is not sequestered by MARCKS. To analyze the specificity of bosutinib treatment further towards protein kinases that are known to modulate MARCKS, we utilized different FRET bioprobes. As expected, bosutinib led to a strong reduction in c-Abl activity. In contrast, activity of PKC, an alternative modulator of MARCKS, was not affected by bosutinib treatment (data not shown).

**Fig. 4:**
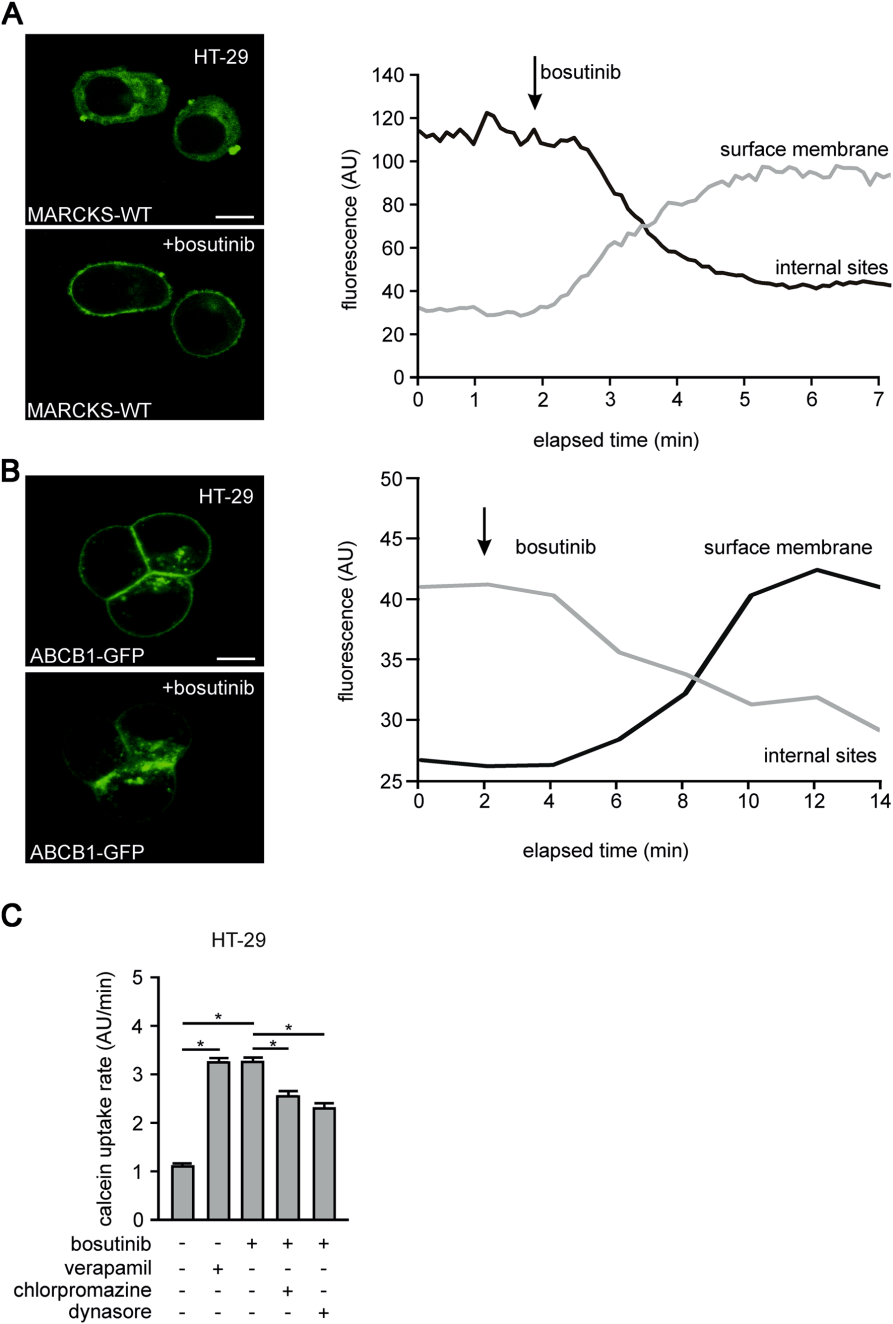
Bosutinib effect on subcellular localization of MARCKS and ABCB1. **a** and **b** Show HT-29 cells transfected with a cDNA encoding fluorescence-tagged MARCKS (MARCKS-GFP) or fluorescence-tagged ABCB1 (ABCB1-GFP), respectively, before and after treatment with bosutinib analyzed via confocal imaging as well as time resolved fluorescence measurements at membrane and internal sites. **c** Depicted are calcein assays of HT-29 cells treated with bosutinib, verapamil and the endocytosis inhibitors chlorpromazine or dynasore as indicated

Next, we evaluated the association between MARCKS function and ABCB1 activity by analyzing the intracellular localization in HT-29 cells. For this purpose, we generated stable cell lines expressing low levels of MARCKS-eGFP or ABCB1-eGFP fusion proteins. Confocal microscopy revealed that MARCKS-eGFP was predominantly localized in the cytosol, whereas ABCB1 was found at the membrane. Notably, upon treatment with bosutinib MARCKS relocalized to the plasma membrane (Fig. 4a). In parallel ABCB1 was translocated to intracellular compartments (Fig. 4b).

**Fig. 5:**
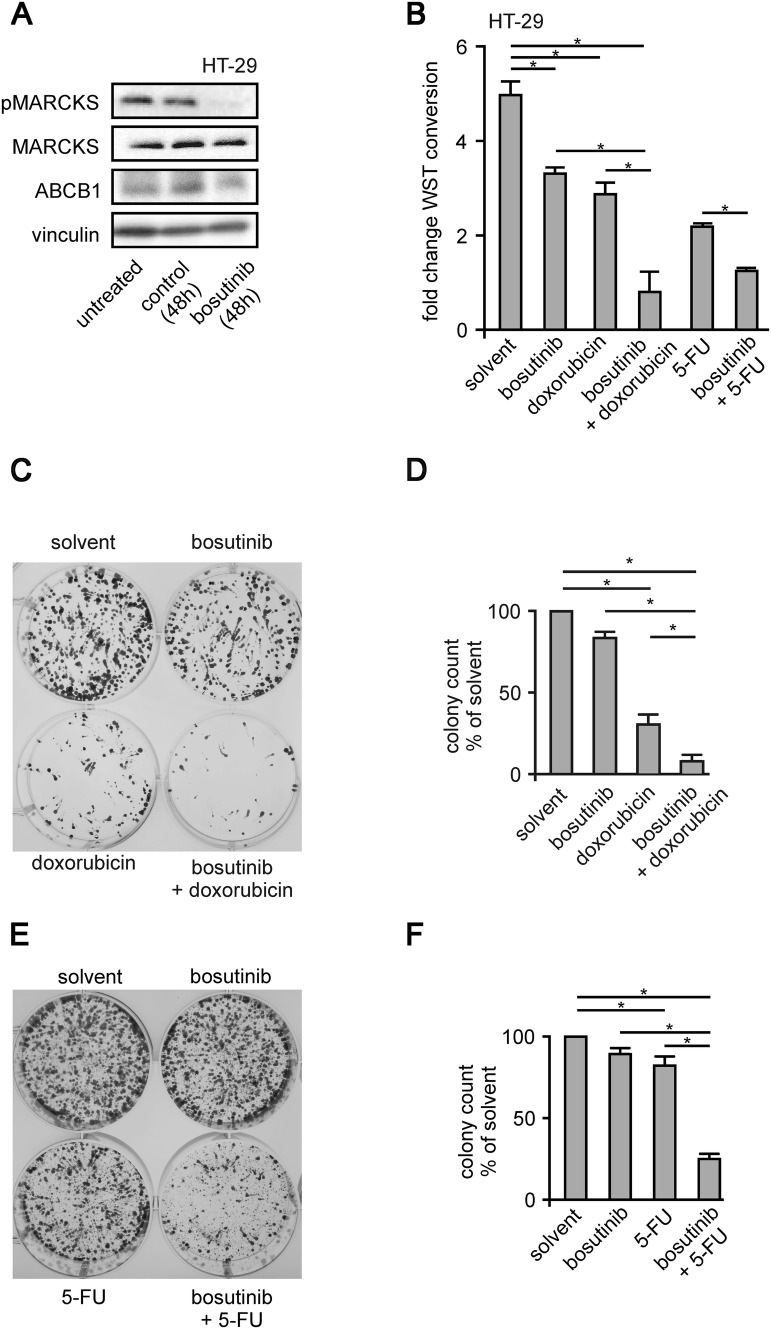
Bosutinib effect on MARCKS phosphorylation and chemoresistance of CRC cells. **a** Shows a typical western blot of HT-29 cells treated with bosutinib for 48 h as indicated. Cells were lysed, harvested, blotted and probed with antibodies against phospho-MARCKS, MARCKS, ABCB1 and vinculin. **b** Depicts WST-1 metabolic activity assays of HT-29 cells after 0 or 5 days of exposure to doxorubicin or 5-fluorouracil treatment as indicated. **c** and **e** [(Zeilenumbruch vor C) and **e**] show Colony forming assays of HT-29 cells treated as indicated, stained with tryphan blue after 2 week incubation with doxorubicin or 5-fluorouracil. Respectively, **d**, **f** statistical quantification of four independent experiments

It is known that the clathrin dependent protein internalization requires the precise removal of PI(4,5)P_2_ from the protein dynamin at the endocytotic region. We wanted to test if ABCB1 inhibitory effects of bosutinib are based on accelerated endocytosis. We employed pharmacological inhibitors against several key players of endocytosis in our calcein assay system. The bosutinib-induced reduction of ABCB1 function was reversed by chlorpromazine (50 µM), a poorly specific inhibitor of clathrin-mediated endocytosis (*n* = 31; *p* = 0.02), or by dynasore (80 µM), a direct inhibitor of dynamin-induced vesicle separation (*n* = 31; *p* = 0.03); (Fig. 4c). We wanted to see if this mechanism resulted in a prolonged reduction of ABCB1 abundance. Here (Fig. 5a), Western-blot experiments revealed that bosutinib treatment led to the significant reduction of ABCB1 expression in HT-29 cells.

### Chemosensitiziation of HT-29 cells upon inhibition of MARCKS phosphorylation

In the light of these findings and given the fact that CRC cell sensitivities towards cytostatics can be dependent on ABCB1, a long-term bosutinib treatment may sensitize HT-29 cells to commonly used chemotherapeutics.

To explore this possibility, we performed cell proliferation assays using the WST-1 tetrazolium dye. We chose doxorubicin and 5-FU as chemotherapeutic agents, because there are either the classical example for ABCB1 function (doxorubicin) or broadly used in the treatment of CRC (5-FU). Both drugs are known for being transported via ABCB1 (Borst and Schinkel 2013; Chufan et al. 2015; Crowley et al. 2010). Upon single treatment of cells with bosutinib, doxorubicin or 5-FU, profound anti-proliferative effects were observed (Fig. 5b). Importantly, the combination of both agents with bosutinib displayed even greater effects, essentially abolishing cell proliferation. This was seen at an even greater magnitude in colony forming assays. Here upon a combination treatment with bosutinib and either doxorubicin or 5-FU reduced the colony numbers by 83% ± 9% (doxorubicin) or 72% ± 12%; (5-FU), respectively (*n* = 6; Fig. 5c–f). Taken together, this demonstrates that sensitivity to chemotherapy treatment is markedly increased or restored by addition of bosutinib, indicating that bosutinib treatment, through its effects in cases of hyperphosphorylated MARCKS, might be a promising therapeutic approach in preventing or overcoming chemotherapy resistance in CRC treatment.

## Discussion

**Fig. 6:**
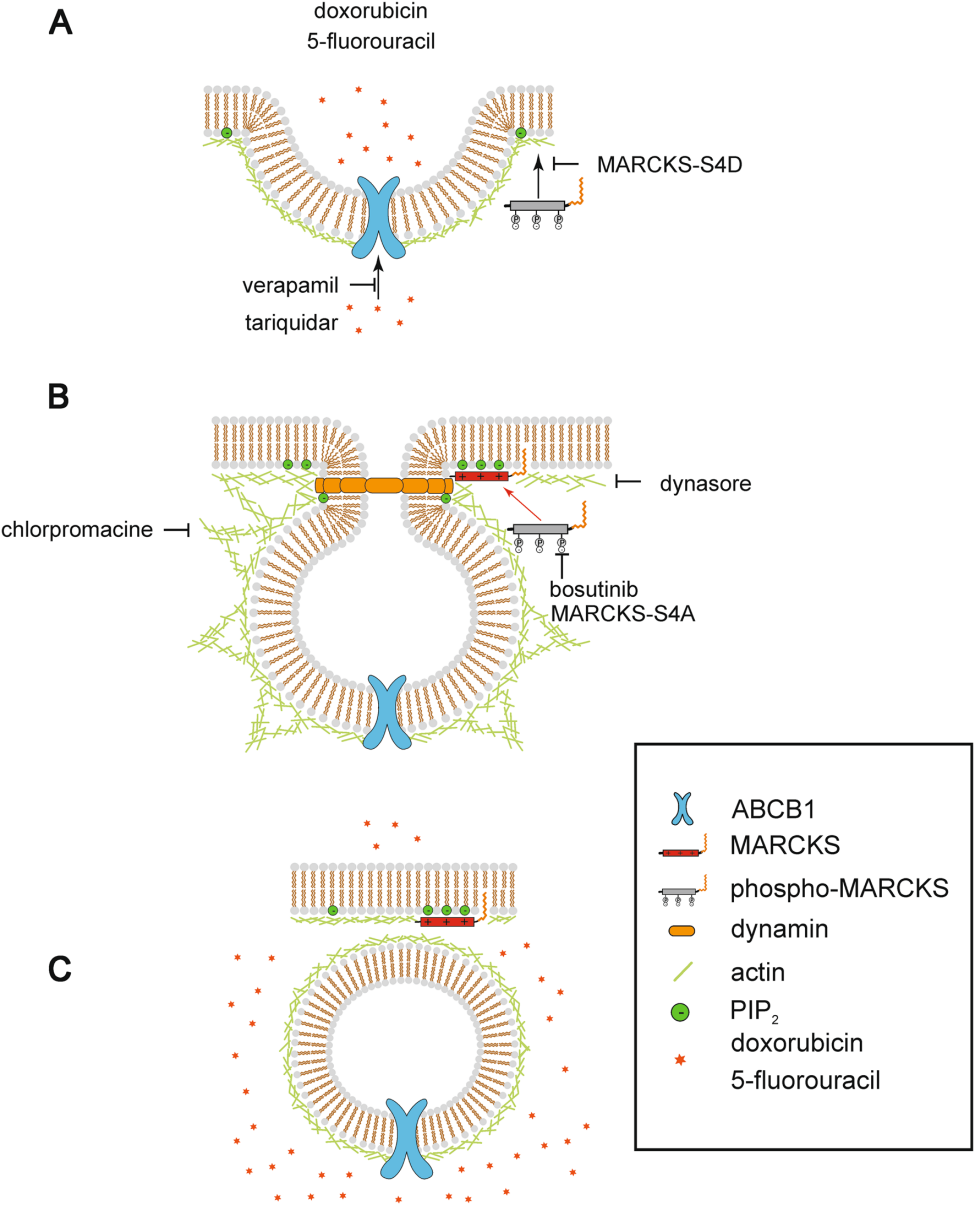
Proposed mechanism of MARCKS—ABCB1 interplay. **a** In a cell of the colorectal carcinoma MARCKS is located in the cytosol in its phosphorylated state. ABCB1 is deregulated and highly present in the plasma membrane. Because of its high abundance, many drugs are transported out of the cell, resulting in enhanced resistance. **b** Bosutinib inhibits the phosphorylation of MARCKS. As a consequence, MARCKS loses its negative charge and binds PI(4,5)P_2_-molecules at the cell membrane. This results in a locally decreased concentration of PI(4,5)P_2_. Due to these changes in membrane organization, the membrane forms a vesicular structure, stabilized by actin. The aggregation of dynamin to the constricted opening helps to pinch off the vesicle with ABCB1 from the plasma membrane. This is facilitated by the—at first—locally elevated concentration of PI(4,5)P_2_ that is subsequently sequestered by MARCKS. **c** The vesicle containing the ABCB1 is internalized. MARCKS remains in its unphosphorylated state at the plasma membrane and continues to sequester PI(4,5)P_2_

In this study, we identify MARCKS as a critical mediator of chemosensitivity, and demonstrate for the first time its loss or its functional disruption by hyperphosphorylation as a pivotal mechanism for increasing the plasma membrane abundance localization and activity of the drug transporter ABCB1. By inhibiting the sequestration of PI(4,5)P_2_ through MARCKS and thereby altered phospholipid homoeostasis, endocytotic internalization of ABCB1 is hindered, thereby leading to tumor cell resistance towards chemotherapy (model in Fig. 6). Concomitantly, we exploited the tyrosine kinase inhibitor bosutinib to reverse MARCKS hyperphosphorylation and to overcome chemotherapy resistance in cellular models of CRC.

Chemotherapy is a cornerstone in the majority of cancer therapies, including CRC. During chemotherapy treatment cycles, tumor subpopulations that successfully exploit resistance mechanisms gain a significant selection advantage (Colabufo 2008), thereby diminishing therapeutic success. In tissues of the digestive tract, the ABCB1 transporter is a naturally occurring safeguard against potentially harmful substances (Borst and Schinkel 2013; Colabufo 2008; Miller 2015), and its aberrant increase in activity has been identified as a major problem in many solid tumors including CRC. This makes ABCB1 an interesting target for the enhancement of tumor therapy, and ABCB1 inhibitor candidates have been shown to dramatically increase the potency of chemotherapeutics in vitro. Although some ABCB1 inhibitors have entered phase III clinical trials (Crowley et al. 2010), so far none has proven effective or safe enough for therapeutic use in humans. This may be due to the fact that ABCB1 is expressed in a wide range of different tissues, making the side effects of a global inhibition too severe, requiring the de-escalation of chemotherapy and thus negating the benefit. For example, the use of the L-type calcium channel blocker verapamil as potent ABCB1 inhibitor showed unsavory side effects like breakdown of the blood–brain barrier as well as severe cardiac problems in clinical trials (Borst and Schinkel 2013; Chufan et al. 2015; Crowley et al. 2010).

To date, our mechanistic insight into how cancer cells can enhance ABCB1 function is still incomplete. Beyond a simple increase in protein expression, gain of function of ABCB1 may involve a change in protein trafficking, leading to increased rates of transporter integration into the plasma membrane or to reduction of its cellular internalization. Most transport processes critically rely on carefully orchestrated changes in the lipid composition of cellular membranes (McLaughlin and Murray 2005; Posor et al. 2015; Roux et al. 2010; Sun et al. 2007), including different species of phosphoinosides. PI(4,5)P_2_ is a high-abundance phospholipid only present at the inner leaflet of the plasma membrane or related membrane structures.

Besides *de novo* synthesis, cells exert control of PI(4,5)P_2_ via capture and release by MARCKS. Due to its biophysical properties, this regulatory protein is not present in the cytosol. In its non-phosphorylated state, the poly-basic protein MARCKS functions as an electrostatic sink that binds to PI(4,5)P_2_. By this means it masks the electrostatic properties necessary for PI(4,5)P_2_ interaction with other signaling molecules, e.g., PI3-kinases. Thus, MARCKS negatively affects a plethora of PI(4,5)P_2_-regulated, tumor relevant processes like proliferation and migration.

Interestingly, recent publications provided evidence of reduced MARCKS activity in CRC being associated with increased malignancy (Bickeböller et al. 2015; Chen et al. 2014, 2015; Chiu 2009; Clarke et al. 1993). In this context, either genetic deletion via frameshift mutations or a cytosolic retention via a hyperphosphorylation of MARCKS was found to correlate with a negative patient outcome (Bickeböller et al. 2015; Chen et al. 2015). Of note, different studies indicate that an excessive percentage of hyperphosphorylated MARCKS is not only prevalent in colorectal cancer but also occurs in hepatocellular carcinoma, lung and breast cancer (Bickeböller et al. 2015; Chen et al. 2015; Yang et al. 2015). Providing a link to chemotherapy, Chen and colleagues could elegantly show that MARCKS hyperphosphorylation occurs as a reaction to cytostatic treatment (Chen et al. 2014, 2015). Our observation of MARCKS deletion or hyperphosphorylation in CRC samples is in line with these reports. Thus, screening for MARCKS expression and/or phosphorylation could be a valuable prognostic marker, also with regard to assessing chemosensitivity.

Our findings demonstrating that MARCKS regulates ABCB1 activity add another facet to the many functions of this protein. Using HT-29 cells as a model for MARCKS hyperphosphorylation, we present herein evidence that the tyrosine kinase inhibitor bosutinib can restore MARCKS function in a tumor context. Bosutinib is a c-Abl inhibitor with an additional effect on Src family kinases. It is mainly used in the treatment of adult patients with chronic myelogenous leukemia. Since bosutinib is already approved for use in cancer patients, our findings that this kinase inhibitor enhances ABCB1 internalization could be of high translational value.

We have shown previously that in a multitude of cell models bosutinib shifts MARCKS into its unphosphorylated form via a PKC-independent mechanism (Jin et al. 2012; Kalwa et al. 2012, 2014; Kalwa and Michel 2011). Using confocal microscopy in HT-29 cells, we found that bosutinib enhances the presence of MARCKS at the membrane, while at the same time, ABCB1 was translocated to internal sites. This rearrangement of ABCB1 suggests an involvement of endocytotic processes. With our observations we propose the following model.

Clathrin is the prototypical molecule involved in endocytosis (Conner and Schmid 2003). The endocytotic mechanism consists of multiple proteins, molecules and their precisely choreographed interactions. The scission of the endocytosis vesicle is the fundamental last step in this process. This complex function largely depends on the interaction between the GTPase dynamin and the phospholipid PI(4,5)P_2_ (Koch and Holt 2012; Posor et al. 2015; Roux et al. 2010; Sun et al. 2007). The removal of PI(4,5)P_2_ is critically important for dynamin function, as it allows the scission process to go through. MARCKS is a protein that captures PI(4,5)P_2_ and hides it away. Consequently, loss of MARCKS or its cytosolic retention leaves the cell with elevated levels of unbound PI(4,5)P_2_. Previous work showed (Echard 2012; Ferguson and de Camilli 2012; Kaksonen et al. 2006; Koch and Holt 2012; Martin 2015; Roux et al. 2010; Sun et al. 2007) that elevated PI(4,5)P_2_ levels result in the continuous formation of abortive endocytotic sites. In this context, endocytosis stops at the level of clathrin coat assembly. This leaves the cell unable to regulate membrane protein abundance through internalization. In our hands, the dynamin 1/2 inhibitor dynasore blocks the bosutinib-induced enhancement in calcein uptake in a similar manner but with greater potency than the poorly specific clathrin inhibitor chlorpromazine. A mechanistically coherent pathway emerges, how MARCKS assists in the completion of the endocytic cycle, thereby allowing the cell to internalize ABCB1. Of note, it has been shown previously that forced overexpression of ABCB1 led to a cellular efflux of bosutinib (Redaelli et al. 2015), raising the question if bosutinib may represent a substrate of ABCB1 and compete with other compounds for its transport capacity. This scenario would represent an alternative explanation for the observed bosutinib-promoted sensitization of CRC cells towards chemotherapy. However, ABCB1 has been demonstrated to be a rather inefficient transporter of bosutinib, which makes it unlikely that the strong sensitizing effects are due to a simple competition for transport capacity. Moreover, MARCKS-negative LoVo cells showed no inhibition of ABCB1 transport activity upon bosutinib treatment, thus excluding a direct bosutinib effect on ABCB1. In contrast, reintroducing wild-type or phosphor-negative MARCKS in LoVo cells significantly decreased ABCB1 transport activity, which was not the case for effector domain-modified or phosphomimicking mutants that are unable to bind and sequester PI(4,5)P_2_. This strongly suggests that bosutinib effects rely on intact MARCKS activity.

## Conclusion

In this study, we have characterized MARCKS as a key factor for the membrane expression of ABCB1 (Fig. 6). Disinhibition of MARCKS function via bosutinib led to increased sensitivity of tumor cells towards chemotherapy, suggesting a therapeutic benefit of bosutinib treatment in CRC chemotherapy.

Importantly, bosutinib should not interfere with normal MARCKS function in nonmalignant cells, where high MARCKS activity represents the normal physiological state. Thus, the combination of chemotherapy with bosutinib may offer significant advantages over previous strategies based on ABCB1 inhibitors, especially with regard to avoiding side effects. Interestingly, a recent study showed that a combination of bosutinib and the thymidylate synthase inhibitor capecitabine, an oral fluoropyrimidine that delivers 5-FU to the tumor, was well tolerated and showed therapeutic promise in a variety of cancers. Thus, further studies regarding the therapeutic potential of bosutinib treatment are warranted. In this context, it is noteworthy that the phosphorylation state of MARCKS is also increased in other tumor entities including carcinomas of the breast, lung or liver (Bickeböller et al. 2015; Chen et al. 2014, 2015; Yamaguchi et al. 2006; Yang et al. 2015). Screening for hyperphosphorylated MARCKS could identify tumors with primary but pharmacologically addressable resistance. Here interfering with MARCKS phosphorylation with the aim of MARCKS re-activation could define a novel avenue in individualized tumor therapies.

## Abbreviations

5-FU: 5-Fluorouracil
ABCB1: ATP-binding cassette subfamily B member 1
AM: Acetoxymethyl
c-Abl: Abelson murine leukemia viral oncogene homolog 1
CCD: Charge-coupled device
CFP: Cyan fluorescent protein
CRC: Colorectal cancer
DAG: Diacylglycerol
DMSO: Dimethyl sulfoxide
ED: Effector domain
FACS: Fluorescence-activated cell sorting
FBS: Fetal bovine serum
FRET: Fluorescence resonance energy transfer
GFP: Green fluorescent protein
IMDM: Iscove’s Modified Dulbecco’s Medium
IP_3_: Inositol-1,4,5-trisphosphate
MARCKS: Myristoylated Alanine-Rich C-Kinase Substrate
MDR1: Multidrug resistance protein 1
P-gp: Permeability glycoprotein
PI3-kinase: Phosphatidylinositol-4,5-bisphosphate 3-kinase
PI(4,5)P_2_: Phosphatidylinositol-4,5-bisphosphate
PKC: Protein kinase C
Src: Proto-oncogene tyrosine–protein kinase sarcoma
TIRF: Total internal reflection fluorescence
WST: Water-soluble tetrazolium salt
YFP: Yellow fluorescent protein
